# Supplementary material for: Pleomorphic effects of three small-molecule inhibitors on transcription elongation by Mycobacterium tuberculosis RNA polymerase
Source: bioRxiv. 2025 Feb 7:2025.02.07.637008. Preprint. [Version 1] doi: 10.1101/2025.02.07.637008 (PMC11839117; doi:10.1101/2025.02.07.637008)
Supplement: Supplement 1 [file NIHPP2025.02.07.637008v1-supplement-1.pdf]

bioRxiv preprint doi: https://doi.org/10.1101/2025.02.07.637008; this version posted February 7, 2025. The copyright holder for this preprint (which was not certified by peer review) is the author/funder, who has granted bioRxiv a license to display the preprint in perpetuity. It is made available under aCC-BY 4.0 International license.

907 **Supplementary Figures**

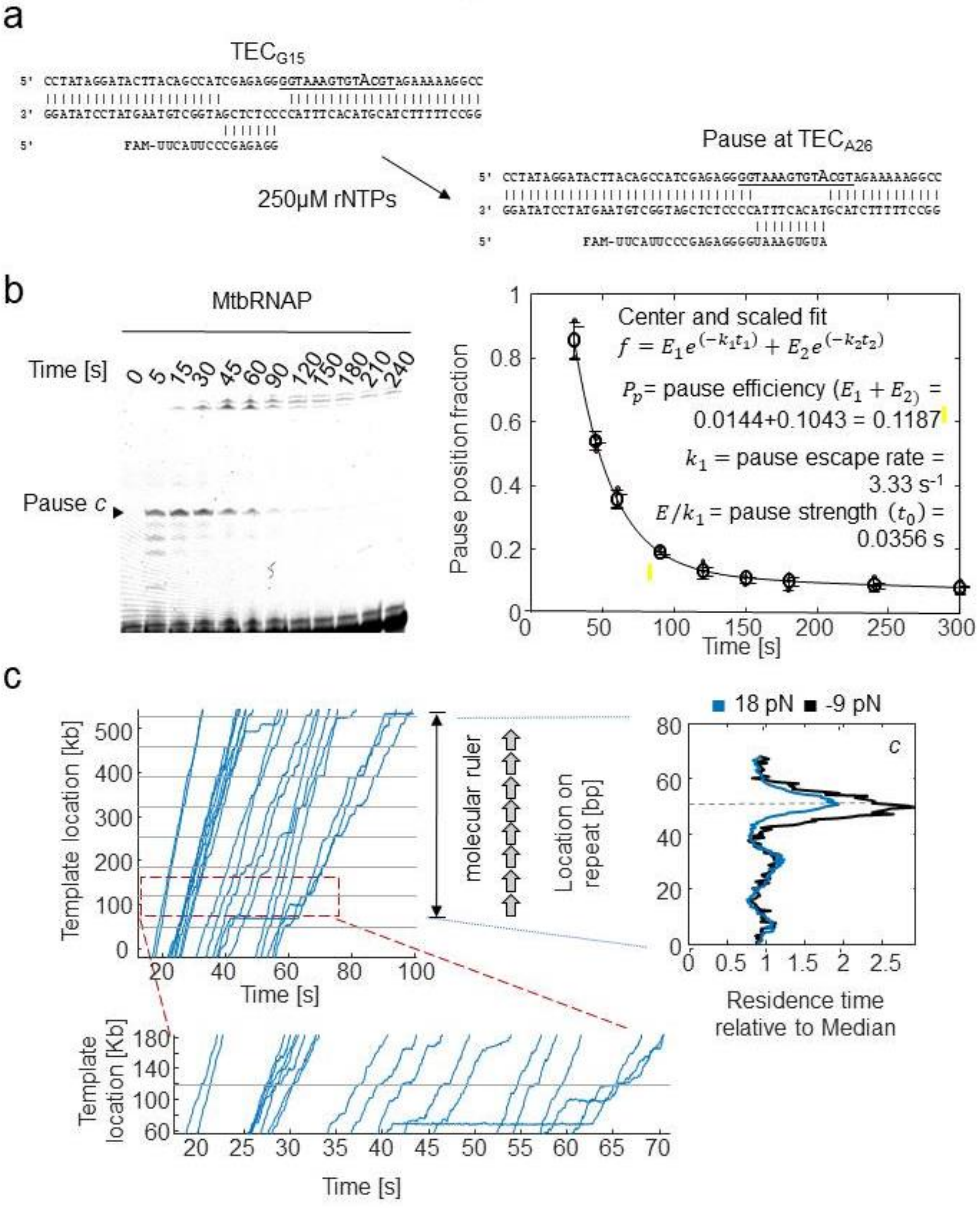

908

bioRxiv preprint doi: https://doi.org/10.1101/2025.02.07.637008; this version posted February 7, 2025. The copyright holder for this preprint (which was not certified by peer review) is the author/funder, who has granted bioRxiv a license to display the preprint in perpetuity. It is made available under aCC-BY 4.0 International license.

Figure S1: The design of a *molecular ruler* to study pausing in MtbRNAP with high-resolution optical tweezers

a) An artificial transcription bubble scheme with a 5'-end labeled RNA with FAM was used for bulk elongation assay. The stalled ternary elongation complex at position G15 (TEC_{G15}) resumes RNA polymerization activity upon adding 250 µM rNTPs.
b) MtbRNAP efficiently recognizes sequence derived from *E. coli* elemental pause *c*. (left) A urea PAGE gel that shows the clearance of the pause site at TEG_{A26} with a mean duration of ~ 27 sec (inverse of the pause escape rate). (right) The quantification of the bands and their fitting to a bi-exponential decay. The extracted parameters indicate that the pause escape rate is kinetically equivalent to *his* pause of EcoRNAP[70].
c) The *Mtb* molecular ruler enables the analysis of sequence dependence pausing by MtbRNAP at the single-molecule level. Example traces are shown on the left, collected at 1mM rNTPs and 18 pN assisting force. Below is a zoom around the designed pause. The RTH on the right shows the strong pausing at 50 bp, and how the strength changes with the direction of the applied force.

Figure S1—Source Data 1: Original gels for Figure S1b, indicating the conditions and relevant bands

Figure S1—Source Data 2: Original files of the gels in Figure S1b.

bioRxiv preprint doi: https://doi.org/10.1101/2025.02.07.637008; this version posted February 7, 2025. The copyright holder for this preprint (which was not certified by peer review) is the author/funder, who has granted bioRxiv a license to display the preprint in perpetuity. It is made available under aCC-BY 4.0 International license.

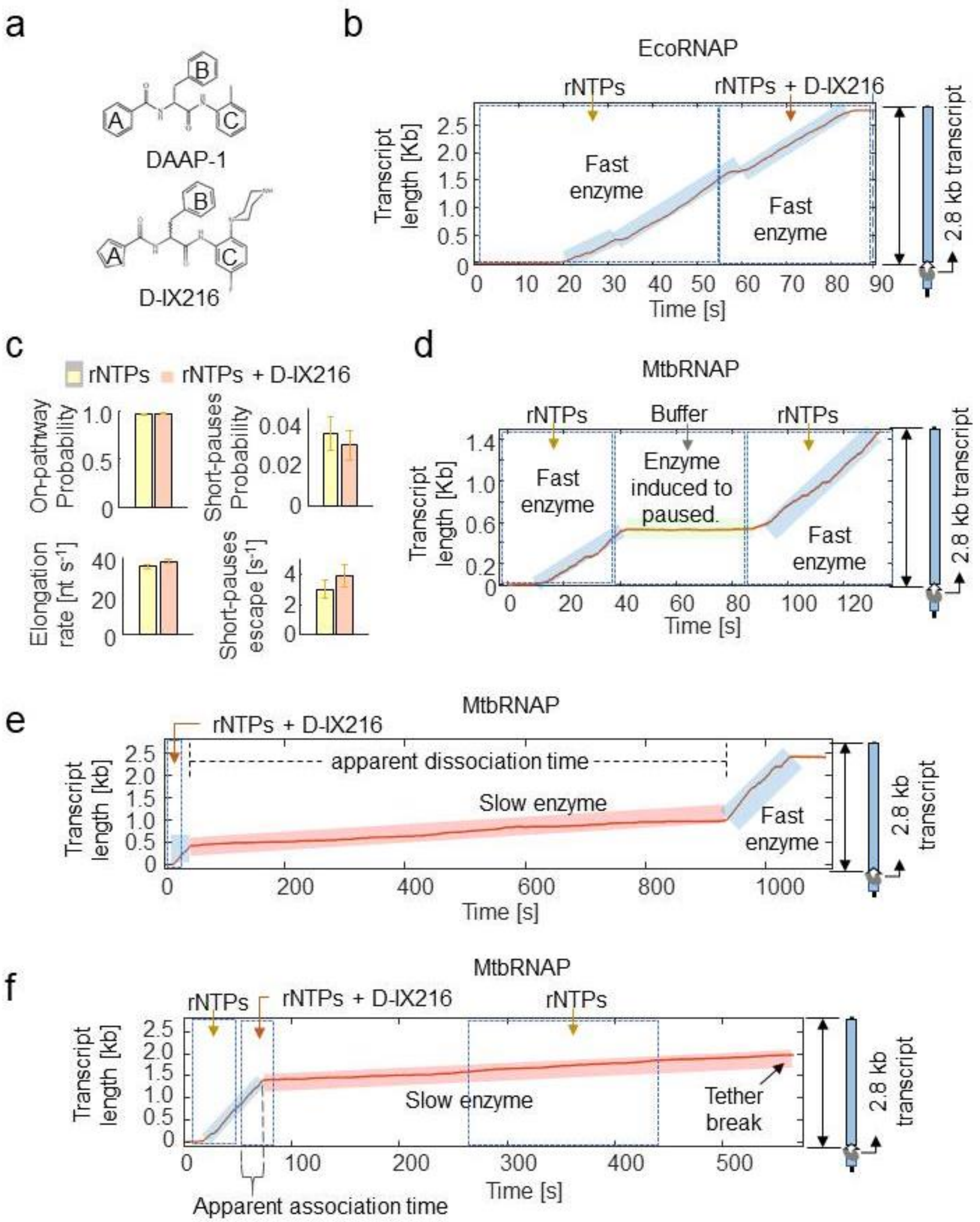

Figure S2: D-IX216 specifically slows down the MtbRNAP transcription

a) The chemical representation of the Nα-aroyl-N-aryl-Phenylalaninamides (AAPs) D-AAP1 and D-IX216 are shown. Rings A and C are modified to increase the affinity of D-IX216 for MtbRNAP[25].

bioRxiv preprint doi: https://doi.org/10.1101/2025.02.07.637008; this version posted February 7, 2025. The copyright holder for this preprint (which was not certified by peer review) is the author/funder, who has granted bioRxiv a license to display the preprint in perpetuity. It is made available under aCC-BY 4.0 International license.

b) D-IX216 does not alter a single EcoRNAP activity. An example trace of a two-shunt experiment shows that 280 nM D-IX216 does not affect EcoRNAP activity. The dotted boxes denote what solution is flowing out of the shunt at what time.

c) The kinetic parameters obtained from DTD analysis of EcoRNAP in the absence and presence of D-IX216 is shown. In this dataset, there were too few events to fit a third exponential (long pauses).

d) This buffer exchange control experiment shows that the RNAP quickly responds to buffer changes. When nucleotides are removed, MtbRNAP immediately pauses until the nucleotides are reintroduced.

e) D-IX216 slows down MtbRNAP rather than pausing or halting it. The trajectory of a single MtbRNAP elongating at constant 18 pN assisting force with 280 nM D-IX216 and saturating nucleotides (~1 mM) shows a slow state that eventually instantly recovers its velocity (~ 20 bp/s). Depending on the condition, up to ~ 41% of polymerases can recover their initial velocity. The dotted boxes show the start and duration of the shunt opening to supplement the inhibitor and nucleotides. The apparent association time (t_a) for D-IX216 binding corresponds to the time between the shunt opening to introduce D-IX216 and the switch's appearance. On the other hand, the apparent D-IX216 dissociation time corresponds to the slow region's lifetime.

f) D-IX216 is still engaged in MtbRNAP elongation. We performed a buffer exchange using two shunts to remove the inhibitor from the affected polymerase. However, this did not immediately restore its global velocity. Towards the end of the trace, the polymerase might detach from the DNA template, leading to tether break events or stalls in 45% and 13% of the polymerases, respectively.

bioRxiv preprint doi: https://doi.org/10.1101/2025.02.07.637008; this version posted February 7, 2025. The copyright holder for this preprint (which was not certified by peer review) is the author/funder, who has granted bioRxiv a license to display the preprint in perpetuity. It is made available under aCC-BY 4.0 International license.

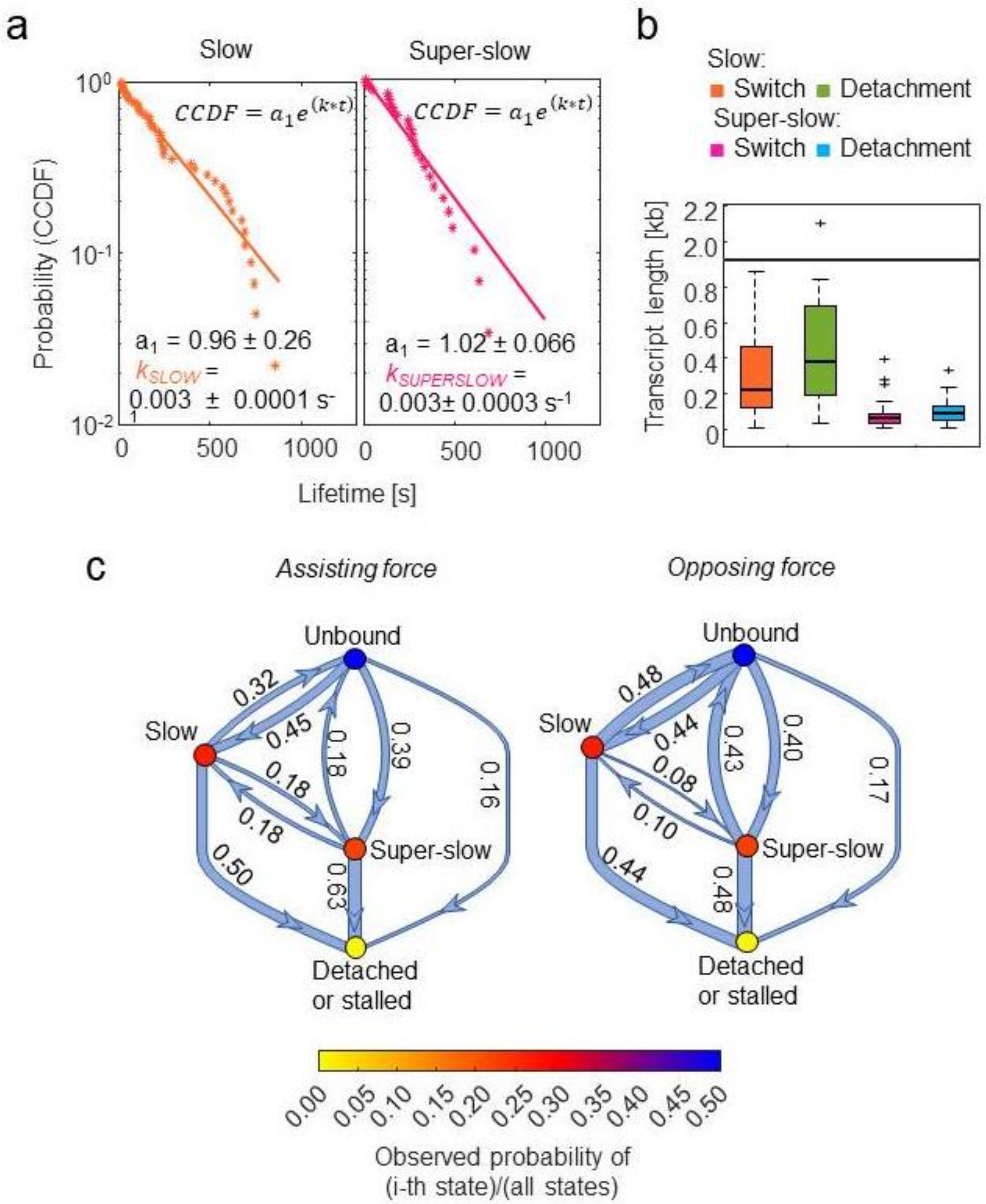

Figure S3: Interconversion between the slow and super-slow inhibited states

a) The CCDFs of lifetimes of the slow and super-slow inhibited states are shown with a fitting to a single exponential decay. The lifetime was measured as the time between switching into that state, until the motor either recovered (fast) or switched into the other state (e.g. a switch from slow to super-slow). Tether-break events were not included.

b) Comparison of the processivity (in terms of kb extended by the elongating polymerase before tether break (template dissociation), or in terms of kb before a switch event of the super-slow and slow inhibited states is shown. Processivity is lower in the super-slow inhibited state than in the slow inhibited state by both

bioRxiv preprint doi: https://doi.org/10.1101/2025.02.07.637008; this version posted February 7, 2025. The copyright holder for this preprint (which was not certified by peer review) is the author/funder, who has granted bioRxiv a license to display the preprint in perpetuity. It is made available under aCC-BY 4.0 International license.

definitions. The data for the bar plots were obtained from combined data of assisting and opposing forces experiments.

c) Transition maps under assisting and opposing force of the different states (fast, slow, and super-slow) are shown. Opposing force increases the probability that the enzyme recovers to the fast (unbound) state compared to assisting force.

bioRxiv preprint doi: https://doi.org/10.1101/2025.02.07.637008; this version posted February 7, 2025. The copyright holder for this preprint (which was not certified by peer review) is the author/funder, who has granted bioRxiv a license to display the preprint in perpetuity. It is made available under aCC-BY 4.0 International license.

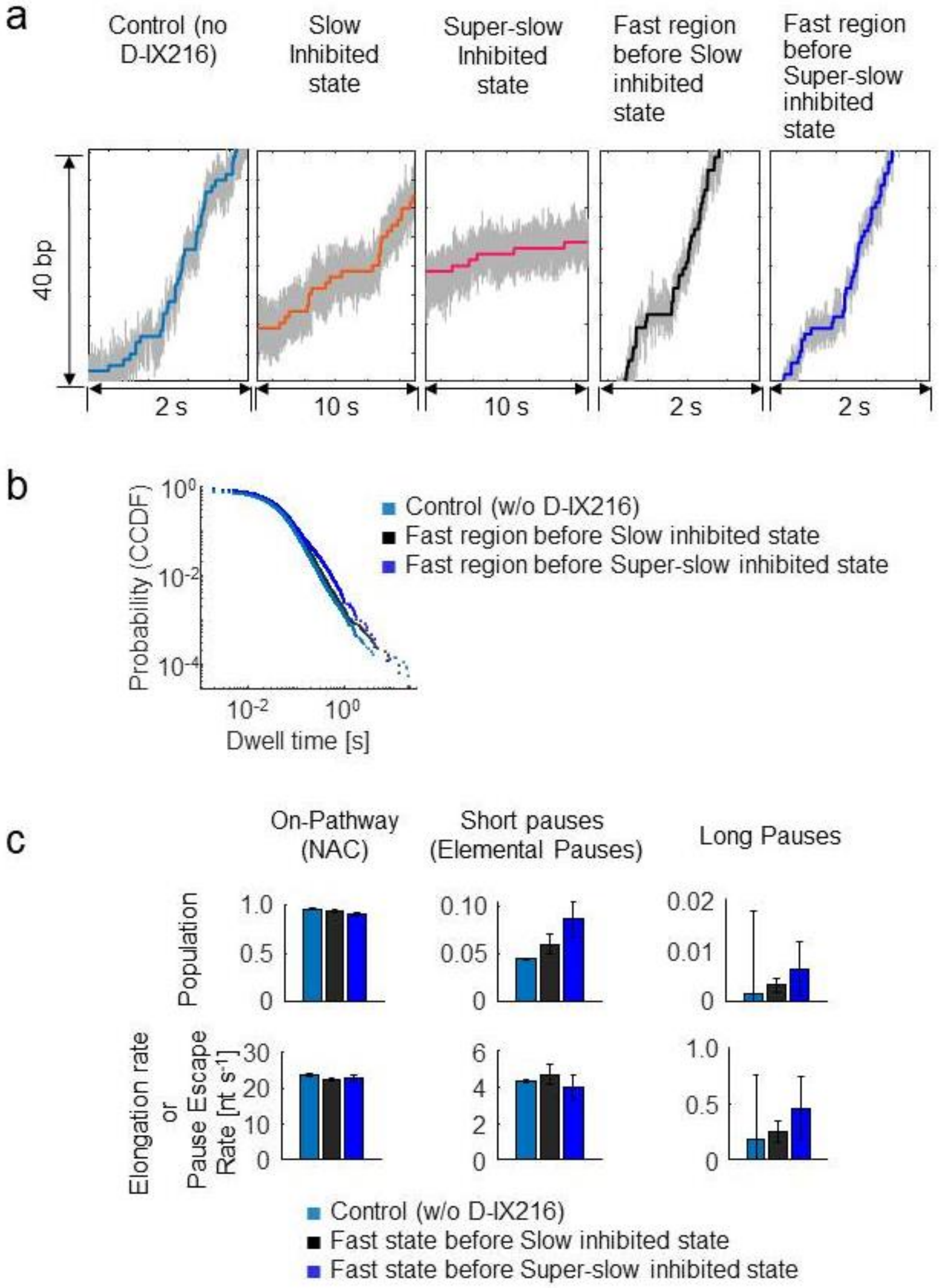

Figure S4: The fast elongating state of MtbRNAP observed in the presence of D-IX216 corresponds to the inhibitor-free state

a) Example fittings of the staircase stepping fitting for different inhibited states of MtbRNAP are shown.

b) The CCDFs of the DTDs for fast elongating polymerases observed with (black and dark blue) and without D-IX216 (cyan).

bioRxiv preprint doi: https://doi.org/10.1101/2025.02.07.637008; this version posted February 7, 2025. The copyright holder for this preprint (which was not certified by peer review) is the author/funder, who has granted bioRxiv a license to display the preprint in perpetuity. It is made available under aCC-BY 4.0 International license.

c) The kinetic parameters from DTD analysis for the enzyme without D-IX216 and the enzyme in the fast state with D-IX216 are shown. No notable differences are found between the inhibitor-free and the fast state with D-IX216.

bioRxiv preprint doi: https://doi.org/10.1101/2025.02.07.637008; this version posted February 7, 2025. The copyright holder for this preprint (which was not certified by peer review) is the author/funder, who has granted bioRxiv a license to display the preprint in perpetuity. It is made available under aCC-BY 4.0 International license.

Fig. S5

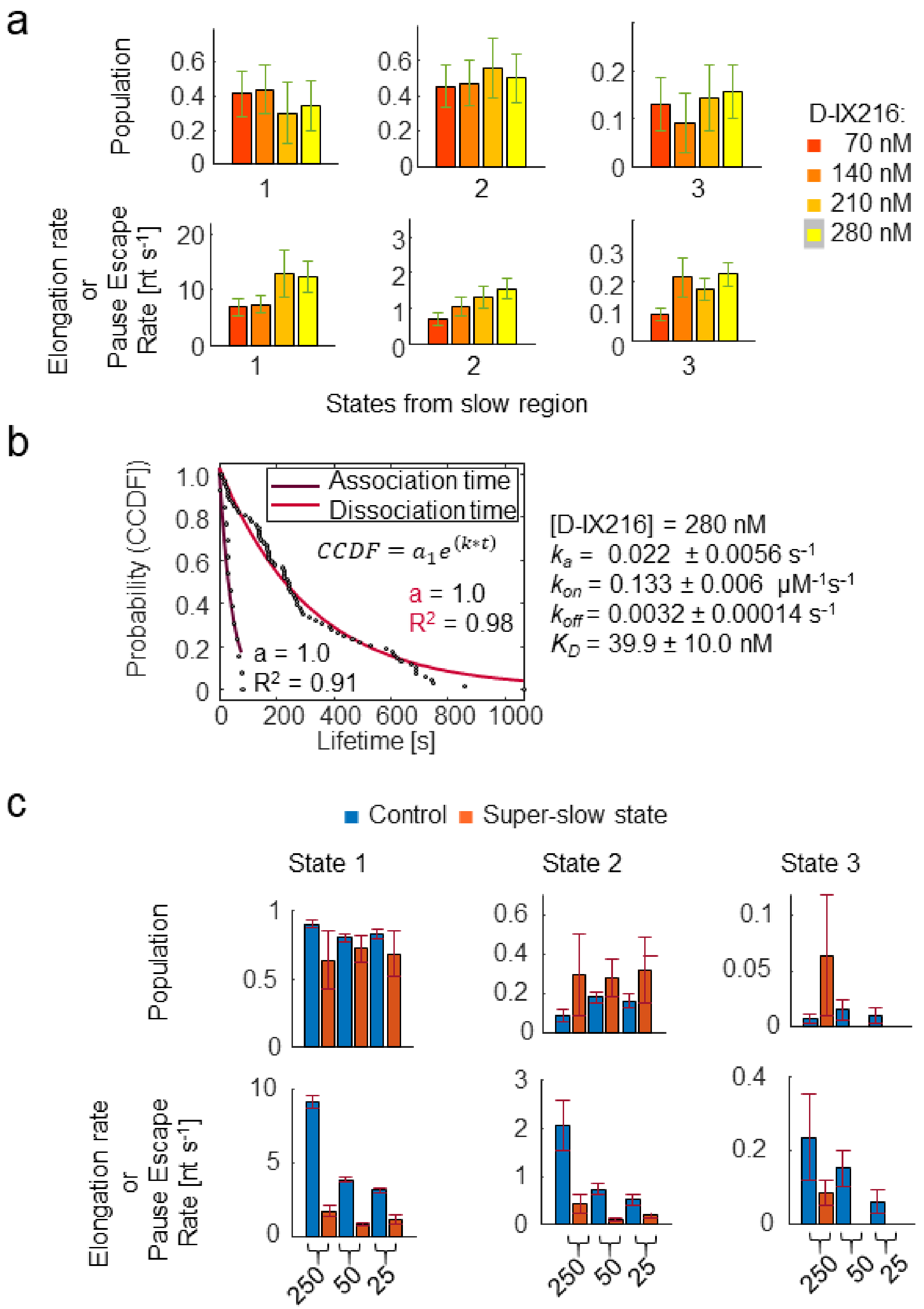

988

bioRxiv preprint doi: https://doi.org/10.1101/2025.02.07.637008; this version posted February 7, 2025. The copyright holder for this preprint (which was not certified by peer review) is the author/funder, who has granted bioRxiv a license to display the preprint in perpetuity. It is made available under aCC-BY 4.0 International license.

Figure S5: Effect of different D-IX216 and nucleotide concentrations on the slow and super-slow inhibited states of MtbRNAP

a) The concentration of D-IX216 did not impact the kinetic parameters obtained by DTD analysis of MtbRNAP in the slow inhibited states. This suggests that a single molecule of D-IX216 causes the conversion into the slow state.
b) Measurement of the dissociation constant (K_D) of D-IX216 for the elongating MtbRNAP. The association and dissociation times obtained from the duration of the fast, slow, and super-slow inhibited states in the single-molecule traces are fit to single-exponentials to obtain k_{on} and k_{off}. Their ratio is K_D.
c) The concentration of rNTPs did not affect the kinetic parameters in the slow inhibited state of MtbRNAP induced by D-IX216. This indicates that the slowing by D-IX216 is not due to a reduction in nucleotide affinity. These experiments were done in semipassive assisting force mode, where the optical traps are moved stepwise to keep the force within a set range. In other words, the traps are held at constant position, causing the force to fall as transcription progresses, until it reaches some lower force limit (in this case, 10pN) after which the traps move apart to raise the force to some upper force limit (in this case, 18pN).

bioRxiv preprint doi: https://doi.org/10.1101/2025.02.07.637008; this version posted February 7, 2025. The copyright holder for this preprint (which was not certified by peer review) is the author/funder, who has granted bioRxiv a license to display the preprint in perpetuity. It is made available under aCC-BY 4.0 International license.

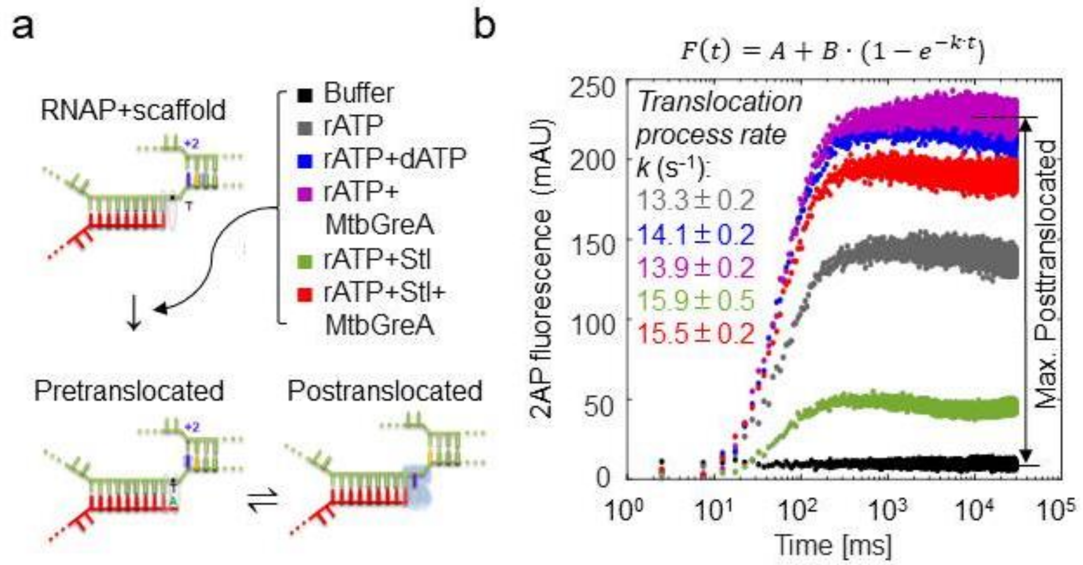

Figure S6: Streptolydigin (Stl) induces reduced post-translocated states in MtbRNAP

a) A bulk assay was performed to measure the translocation of RNAP by one nucleotide using the Mtb ternary elongation complex. The technique involved real-time measurement of the fluorescent enhancement at 375 nm of a nitrogenous base analog, 2-aminopurine[52]. This analog was positioned on the template strand of the DNA-RNA hybrid, initially at position +2, and then moved towards position +1 due to the RNAP translocation process. The workflow included the use of a fluorescent artificial bubble to measure the effect of Stl on the translocation state of MtbRNAP. After the RNAP incorporated one rATP, the translocation state was measured by the dye's fluorescence in the following base in the template DNA. When the base was paired, fluorescence was low due to quenching; when the base was unpaired, such as in the post-translocated state, the fluorescence was high. The assay conditions consisted of transcription buffer and 100 µM rATP, along with either 100 µM dATP, 0.25 µM MtbGreA, 7.5 µM Stl, or 0.25 µM MtbGreA / 7.5 µM Stl.

b) The comparison of different conditions of the translocation trace of the MtbRNAP in the presence of cognate nucleotide (rATP) and Stl shows that the inhibitor allows only ~ 20% of the MtbTEC to attain the post-translocated state (green dots). The addition of MtbGreA was used as a positive control for post-translocation.

bioRxiv preprint doi: https://doi.org/10.1101/2025.02.07.637008; this version posted February 7, 2025. The copyright holder for this preprint (which was not certified by peer review) is the author/funder, who has granted bioRxiv a license to display the preprint in perpetuity. It is made available under aCC-BY 4.0 International license.

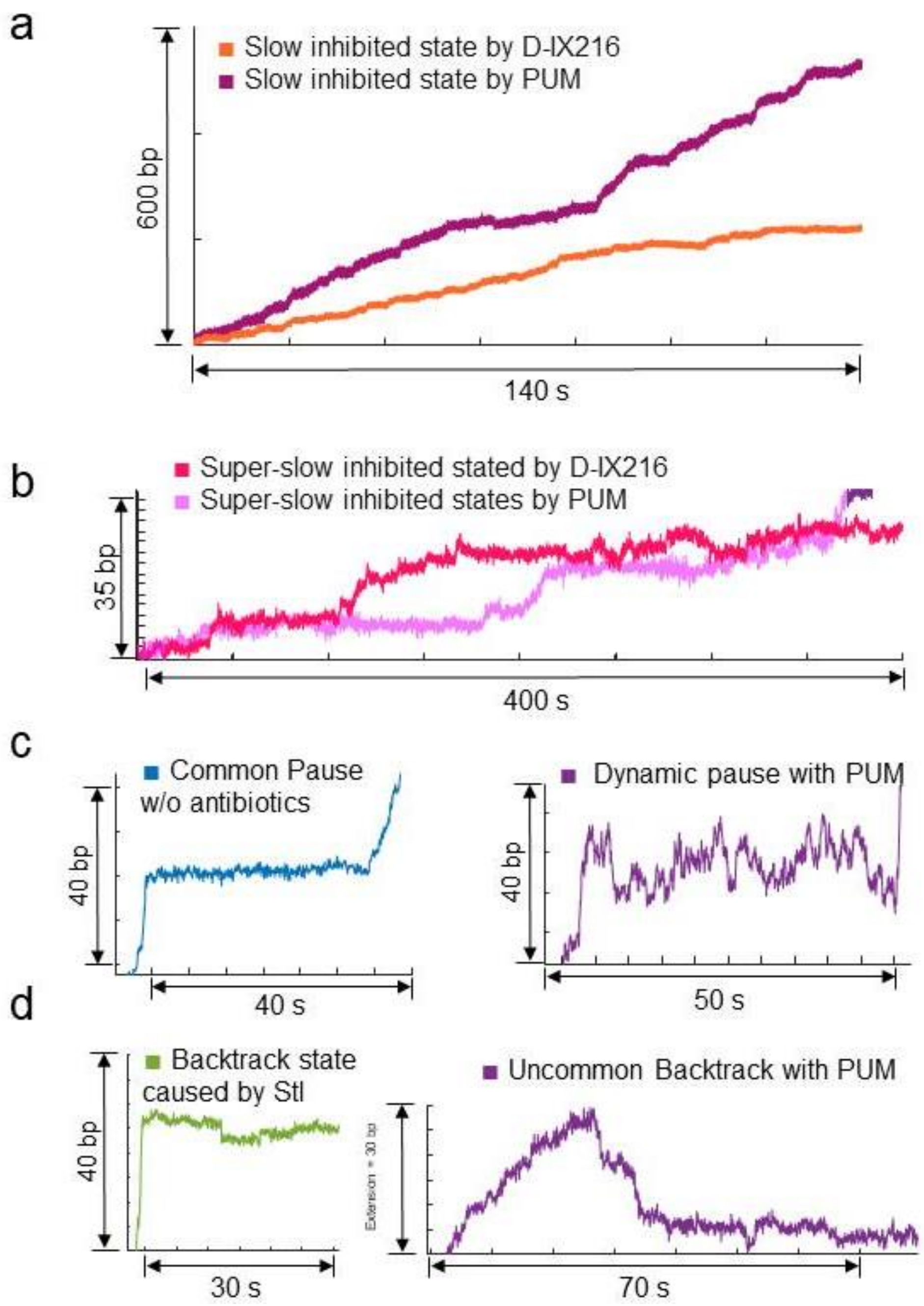

Figure S7: Comparison of events found in transcription with antibiotics

a) Example traces in the slow inhibited state caused by PUM and D-IX216 are shown.
b) Example traces in the super-slow inhibited state caused by PUM and D-IX216 are shown.
c) Comparison of a common pause obtained in the absence of inhibitor versus a dynamic pause caused by PUM is shown.
d) Comparison of a backtrack event observed in the presence of Stl compared to a rare non-traditional backtrack event induced by PUM is shown.
